# Effect and Mechanism of TL1A Expression on Epithelial-Mesenchymal Transition during Chronic Colitis-Related Intestinal Fibrosis

**DOI:** 10.1155/2021/5927064

**Published:** 2021-06-25

**Authors:** Jia Wenxiu, Yang Mingyue, Han Fei, Luo Yuxin, Wu Mengyao, Li Chenyang, Song Jia, Zhang Hong, David Q. Shih, Stephan R. Targan, Zhang Xiaolan

**Affiliations:** ^1^Department of Gastroenterology, The Second Hospital of Hebei Medical University, Hebei Key Laboratory of Gastroenterology, Hebei Institute of Gastroenterology, No. 80 Huanghe Road, Yuhua District, Shijiazhuang, Hebei, China; ^2^F. Widjaja Foundation Inflammatory Bowel and Immunobiology Research Institute, Cedars-Sinai Medical Center, Los Angeles, CA, USA

## Abstract

**Background and Aims:**

Recent evidences reveal that epithelial to mesenchymal transition (EMT) exacerbates the process of intestinal fibrosis. Tumor necrosis factor-like ligand 1A (TL1A) is a member of the tumor necrosis family (TNF), which can take part in the development of colonic inflammation and fibrosis by regulating immune response or inflammatory factors. The purpose of this study was to elucidate the possible contribution of TL1A in onset and progression of intestinal inflammation and fibrosis through EMT.

**Methods:**

Colonic specimens were obtained from patients with inflammatory bowel disease (IBD) and control individuals. The expression levels of TL1A and EMT-related markers in intestinal tissues were evaluated. Furthermore, the human colorectal adenocarcinoma cell line, HT-29, was stimulated with TL1A, anti-TL1A antibody, or BMP-7 to assess EMT process. In addition, transgenic mice expressing high levels of TL1A in lymphoid cells were used to further investigate the mechanism of TL1A in intestinal fibrosis.

**Results:**

High levels of TL1A expression were detected in the intestinal specimens of patients with ulcerative colitis and Crohn's disease and were negatively associated with the expression of an epithelial marker (E-cadherin), while it was positively associated with the expression of interstitial markers (FSP1 and *α*-SMA). Transgenic mice with high expression of TL1A were more sensitive to dextran sodium sulfate and exhibited severe intestinal inflammation and fibrosis. Additionally, the TGF-*β*1/Smad3 pathway may be involved in TL1A-induced EMT, and the expression of IL-13 and EMT-related transcriptional molecules (e.g., ZEB1 and Snail1) was increased in the intestinal specimens of the transgenic mice. Furthermore, TL1A-induced EMT can be influenced by anti-TL1A antibody or BMP-7 *in vitro*.

**Conclusions:**

TL1A participates in the formation and process of EMT in intestinal fibrosis. This new knowledge enables us to better understand the pathogenesis of intestinal fibrosis and identify new therapeutic targets for its treatment.

## 1. Introduction

In patients with inflammatory bowel disease (IBD), recurrent intestinal inflammation triggers mucosal healing reactions, leading to extracellular matrix (ECM) deposition in the intestine to form intestinal fibrosis [1]. As many as one-third of patients with Crohn's disease (CD) develop end-stage fibrotic disease characterized by stenosis and organ failure [2, 3], and 80% of cases require surgical resection of the fibrotic intestinal tissue. Unfortunately, the recurrence rate is as high as 70% [4]. Ulcerative colitis (UC) has long been believed to be a nonfibrotic disease; however, recent studies have found a certain degree of submucosal fibrosis in almost all colon resection specimens from patients with UC [5–7]. Identifying effective treatment for intestinal fibrosis-induced by IBD has become the focus of investigations worldwide.

Myofibroblasts are important effector cells for the deposition of ECM in intestinal fibrosis, and their sources are varied [8]. Exploration into the origins of myofibroblasts may provide an opportunity to develop effective treatments in intestinal fibrosis. An increasing number of studies indicate that epithelial to mesenchymal transition (EMT) is involved in intestinal fibrosis [9]. During this process, epithelial cells lose polarity as well as tight junctions, transform into interstitial cells, and produce a large amount of ECM deposited in the intestine, resulting in intestinal lumen narrowing [10]. Experiments with a transgenic mouse model with epithelial-driven expression of fluorescent reporters, indicate that about one-third of FSP1^+^ fibroblasts are derived from intestinal epithelial cells [11]. EMT can also be observed *in vitro*. For example, rat intestinal epithelial cells (IEC-6) displayed an irregular polygonal shape in control medium but obtained a spindle-shaped morphology after exposure to TGF-*β*1 for seven days [11]. Another study showed that parathyroid hormone-like hormone (PTHLH) was capable of increasing vimentin and FSP1 expression and reducing E-cadherin expression in a concentration- and time-dependent manner. Furthermore, exposure to PTHLH leads to collagen deposition [12]. All of these studies suggest that EMT may be an important mechanism in the development of intestinal fibrosis.

The process of EMT is regulated by a complex cell signaling pathway and gene regulation. The TGF-*β*1/Smad3 pathway is the most classical pathway involving TGF-*β*1 [13]. However, exploring when and how it occurs may lead to effective treatment strategies for intestinal fibrosis. Genome-wide association studies (GWAS) have emphasized that the gene encoding tumor necrosis factor-like ligand 1A (TL1A) is associated with susceptibility to UC and CD and is upregulated in both UC and CD patients [14–16]. In recent years, studies have shown that TL1A participates in the development of intestinal inflammation and fibrosis [17, 18]. Moreover, mice with high expression of TLIA can develop spontaneous ileitis, proximal colitis, and even fibrosis, which is associated with elevated levels of fibrotic factor interleukin-13 (IL-13). Elevated secretion of IL-13 is tightly associated with the expression of TL1A in the transgenic mouse model described above [19, 20], and the fourth European Crohn's and Colitis Organisation (ECCO) guidelines state that the TGF-*β*1/Smad3 pathway activated by IL-13 is a central process in the formation of intestinal fibrosis [21].

Further study has shown that in colitic mice with adoptively transferred T cells, intestinal inflammation and fibrosis could be alleviated with the treatment of anti-TL1A antibodies, which reduce the expression of *α*-SMA and vimentin and inhibit the TGF-*β*1/smad3 pathway [22]. Recombinant human bone morphogenetic protein-7 (BMP-7), which belongs to the TGF-*β* superfamily, reverses TGF-*β*1-induced EMT in intestinal fibrosis both *in vitro* and *in vivo* [11].

Therefore, we hypothesized that TL1A promotes intestinal fibrosis by inducing EMT. In this study, we evaluated the correlation between TL1A expression and changes in EMT-related markers in patients with UC or CD. Furthermore, wild-type (WT) mice and TL1A transgenic (Tg) mice were used to establish a chronic colitis-associated intestinal fibrosis model. Moreover, HT-29 cells were stimulated with TL1A, anti-TL1A antibodies, and BMP-7, and the changes in EMT-related markers were evaluated. The effects and mechanisms of TL1A on EMT are discussed in order to bring forward new theoretical bases for the treatment of intestinal fibrosis.

## 2. Methods

### 2.1. Human Tissues

Twelve patients with UC (male/female: 6/6) and ten patients with CD (male/female: 4/6) were enrolled in this study from the Second Hospital of Hebei Medical University (Shijiazhuang, China). The diagnosis of IBD was based on standard clinical, endoscopic, radiological, and histological findings. Normal colonic samples were taken from 8 control individuals (male/female: 4/4), who underwent colonoscopy for other reasons and were found to be normal in examination and histology. The clinical characteristics and inflammatory markers of the three groups are shown in Tables 1 and 2. The age, sex, and other relevant markers of the three groups were matched. This study has been approved by the Ethics Committee of Hebei Medical University.

### 2.2. Mice and Treatment

LCK-CD2-TL1A-GFP-transgenic (Tg) mice overexpressing TL1A in lymphocytes, and wildtype (WT) mice applied in this study, were in the C57BL/6J genetic background. The Tg mice were brought from American Cedars-Sinai Medical Center and Immunology Research Center. All mice were matched by age (8–10 weeks), weight (20–22 g), and sex (female). The animals were housed under specified pathogen-free conditions. The genotypes of the mice used in this study were confirmed through PCR (Supplementary Figure 1). There were four experimental groups: the control/WT group (*n* = 8), the control/Tg group (*n* = 8), the dextran sodium sulfate (DSS)/WT group (*n* = 8), and the DSS/Tg group (*n* = 10). A chronic colitis-related intestinal fibrosis model was generated by the administration of DSS for three cycles. One cycle consisted of 2% DSS in drinking water for seven days followed by normal drinking water for two weeks. All experiments were carried out in accordance with the Ethics Committee of Hebei Medical University.

### 2.3. Evaluation of the Severity of Intestinal Inflammation

The general condition of the mice was observed daily, and the body weights were measured every four days. On the last day of modeling, disease activity index (DAI) scores were performed based on mouse body weight, stool characteristics, and fecal occult blood to assess colonic inflammation [23]. After the mice were euthanized, the colon was removed to record the length and weight, and used for further analyses. Tissue sections of 4 *μ*m were cut from formalin-fixed paraffin-embedded colon tissue blocks and were dewaxed in xylene and rehydrated in graded alcohol washes, then stained with hematoxylin and eosin (H&E) staining to assess histopathological inflammation. Macroscopic scoring and microscopic scoring [24] were used to assess the histological damage. To further evaluate inflammatory cell infiltration, myeloperoxidase (MPO) activity was detected using the Myeloperoxidase Activity Assay Kit (Nanjing Jiancheng Bioengineering Institute, China).

### 2.4. Evaluation of Fibrosis and Chronic Wound Healing

Sirius red staining was conducted to evaluate collagen deposition in colonic tissue specimens embedded in paraffin. Furthermore, *Image-Pro Plus* software (IPP 6.0) was used to evaluate the percent of fibrotic area in IBD and control patients [12]. And fibrosis scoring criteria [25] (Supplementary Table 1) was used to evaluate the fibrosis level in mice. Next, the relative minimal and maximal thickness of submucosa were also calculated in mice [26]. In addition, a hydroxyproline assay was performed to assess collagen content in the colon tissues.

### 2.5. Cell Culture and Cell Viability Assay

The human HT-29 cell line was obtained from the Chinese Academy of Sciences Cell Bank (Shanghai, China) and cultured in McCoy's 5A medium with 10% fetal bovine serum, penicillin (100 units/ml), and streptomycin (100 *μ*g/ml). Cells were grown at 37°C in a 5% CO_2_ atmosphere. Cell Counting Kit-8 assay (CCK-8) was used to detect cell viability. HT-29 cells were seeded 100 *μ*L per well at a density of 1 × 10^4^ cells/well in 96-well plates. When the cell growth reaches logarithmic phase, serum-free media with different concentrations (0 ng/mL, 20 ng/mL, 50 ng/mL, 100 ng/mL, and 150 ng/mL) of TL1A replaced the complete culture medium. After incubating for 12, 24, 48, or 72 h, 10 *μ*L CCK-8 was added to each well and incubated at 37°C for an additional 1 h. Then, the optical densities (OD) under 450 nm were recorded. Based on the results (Supplementary Figure 2), a concentration of 50 ng/mL of TL1A was applied in subsequent experiments.

### 2.6. Immunofluorescence Staining

Colonic tissues and HT-29 cells were treated with 0.5% Triton X-100 for permeability. After blocking with 10% goat serum, sections were incubated with primary antibodies (E-cadherin and FSP1) overnight at 4°C. The sections were then stained with secondary antibodies conjugated with either FITC or Cy3. Nuclei appeared blue with 4′6-diamidino-2-phenylindole (DAPI) staining (Beyotime, Shanghai, China). A laser scanning confocal microscope FV12-IXCOV (Olympus, Japan) were used to obtain images. The mean optical density of images was calculated through Image-Pro Plus software to analyze the positive expression.

### 2.7. Immunohistochemical Staining

Paraffin-embedded mucosal biopsy specimens and mice colonic tissues (4 *μ*m thick sections) were incubated in antigen retrieval solution for 10 min at 95°C. After cooling at room temperature, peroxidases were inhibited with 3% H_2_O_2_ for 10 minutes. Sections were then treated with 10% goat serum for 30 minutes before incubating with primary antibodies (*β*-catenin, E-cadherin, *α*-SMA, FSP1, IL-13, TGF-*β*1, Smad3, Snail, and ZEB1). After washing with PBS three times for 10 minutes, sections were incubated with secondary antibodies for 30 minutes at room temperature. Then, according to the manufacturer's protocol, sections were incubated with the DAB detection kit, then stained with H&E. Three fields of view (400x) of each slice are calculated through Image-Pro Plus software to analyze the positive expression. We obtained the mean optical density value of each group and produced a histogram to show the results.

### 2.8. Western Blot

Proteins from the WT and Tg mouse colonic specimens and HT-29 cells were extracted and concentrations were quantified by the BCA method. Total protein was separated on an SDS-PAGE gel system, then transferred to a PVDF membrane (0.22 *μ*m, Millipore Corp., Billerica, MA, USA). The blot with separated proteins was incubating with primary antibodies (against collagen I, collagen III, *β*-catenin, E-cadherin, *α*-SMA, FSP1, IL-13, TGF-*β*1, Smad3, Snail1, ZEB1, and GAPDH) overnight at 4°C. After washing with Tris-buffered saline-Tween (TBST), the membranes were incubated with fluorescently labeled secondary antibody for 1 h at room temperature. The developed protein bands were quantified using ImageJ and normalized with GAPDH internal controls.

### 2.9. Real-Time Reverse Transcription-Polymerase Chain Reaction (RT-PCR)

Total RNA was extracted from WT and Tg mouse colonic tissues or HT-29 cells following the manufacturer's guidelines and reversed transcribed into cDNA by PrimeScript qRT-PCR Kit (TaKaRa, China). The RT-PCR analysis was performed using SYBR® GreenER™ qPCR SuperMix kit (Invitrogen, Carlsbad, CA) on a 7500 Real-time system (Applied Biosystems, USA). Primer sequences are listed in Supplementary Table 2. GAPDH was used as an internal standard. Data were calculated by the comparative cycle threshold (CT) (2^−ΔΔCT^) method.

### 2.10. Quantification of Cytokines by Enzyme-Linked Immunosorbent Assay (ELISA)

According to manufacturer's instructions, the expression levels of IL-13 and TGF-*β*1 in serum of WT mice and Tg mice were detected using an ELISA kit (R&D, Minneapolis, USA). Changes in absorbance were determined by spectrophotometry at 450 nm wavelength.

### 2.11. Statistical Analysis

Statistical analysis was performed using IBM SPSS Statistics 22.0 (SPSS Inc., Chicago, IL, USA). Data were represented as mean ± standard deviation (SD). The Kolmogorov-Smirnov test was used to test the normal distribution of quantitative data. If the data were normally distributed, one-way ANOVA and the Student-Newman-Keuls (SNK) post hoc tests were used to determine the statistical significance between multiple groups; if not, the Kruskal-Wallis test and the Nemenyi post hoc test were used. The relationship between continuous variables were represented by the Pearson correlation analysis. Differences were noted as significant at ^∗^*P* < 0.05, ^∗∗^*P* < 0.01, and ^∗∗∗^*P* < 0.001.

## 3. Results

### 3.1. Evidence for Intestinal Fibrosis and EMT in Patients with IBD

**Figure 1:**
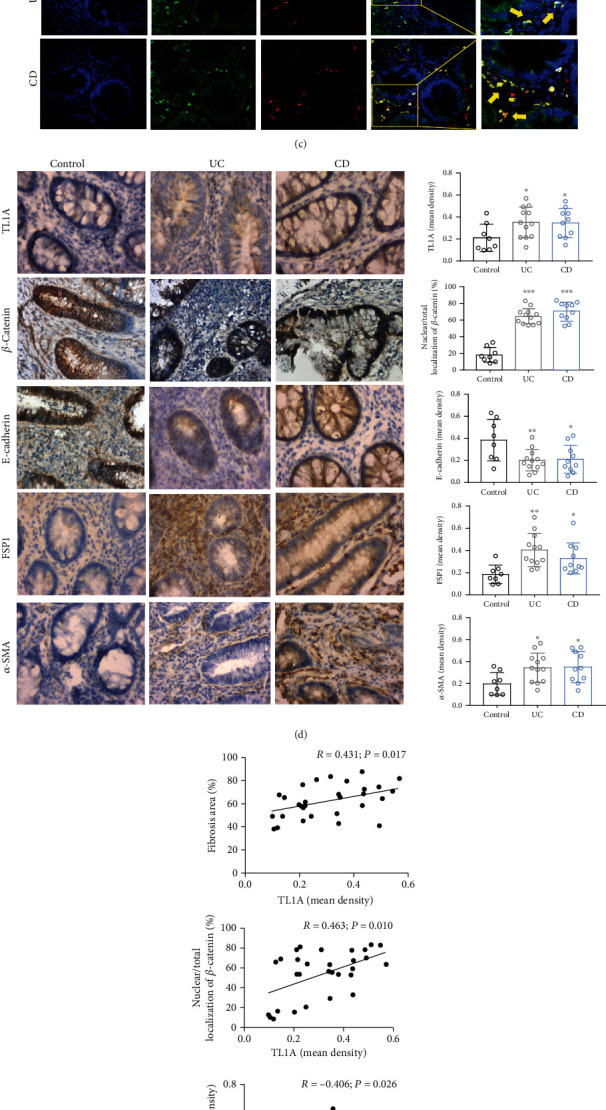
TL1A in colon tissue of patients with ulcerative colitis (UC) and Crohn's disease (CD) correlates with fibrosis score and EMT-related markers. Colon tissues from CD patients (*n* = 10), UC patients (*n* = 12), and controls (*n* = 8) were included. (a, b) Representative microscopic images of hematoxylin and eosin- (H&E-) stained paraffin-embedded colonic tissue samples (100x), and the quantitative determination of fibrosis area based on Sirius red staining (100x). (c) Immunofluorescence double staining. E-cadherin (green), *α*-SMA (red), and colocalization (yellow) in colon tissue samples of IBD patients and controls (400x). DAPI stains nuclei (blue). (d) Immunohistochemical staining of *β*-catenin, TL1A, E-cadherin, FSP1, and *α*-SMA in colon tissues of controls and IBD patients (400x), and mean density of nuclear localization of *β*-catenin and expressions of TL1A, FSP1, and *α*-SMA were increased in the UC and CD groups; however, the mean density of E-cadherin was decreased in the UC and CD groups. (e) Pearson's correlation and linear analysis. Data were given as mean ± standard deviation (SD). As compared to the control group: ^∗^*P* < 0.05, ^∗∗^*P* ≤ 0.01, and ^∗∗∗^*P* ≤ 0.001.

Inflammation destroys the gastrointestinal epithelial barrier, and barrier dysfunction, in turn, causes further spread of inflammation. We collected serum and intestinal samples from 8 controls and 12 UC and 10 CD patients for this study. Serum inflammation marker high-sensitivity C-reactive protein (hsCRP), erythrocyte sedimentation rate (ESR), and white blood cell (WBC) were increased in IBD patients compared with the control group (Table 2). Further, H&E staining revealed that the colonic mucosa was intact in the control group, while erosions and ulcers appeared in the IBD group (Figure 1(a)). Sirius red staining, applied to evaluate collagen deposition and degree of fibrosis in UC and CD intestinal mucosa, revealed substantial collagen deposition as well as fibrotic alterations in the colon tissues of CD and UC patients compared with controls (Figure 1(a)). Fibrosis area was significantly higher in the UC and CD groups than in the control group (Figure 1(b)). To verify the occurrence of EMT, double-label immunofluorescence staining was performed to determine the colocalization of E-cadherin and FSP1. There were more double-positive cells detected in the colon tissues of UC and CD patients than in the control group (Figure 1(c)).

### 3.2. TL1A Expression Was Markedly Increased in IBD Colon and Correlated with Degree of Fibrosis and EMT-Related Markers

To explore the possible contribution of TL1A in IBD, the expression of TL1A was detected in colonic tissues from controls and patients with UC and CD by immunohistochemical staining. *β*-Catenin membrane staining in IBD patients was weaker than the control group, and more cells have nuclear staining, indicating that *β*-catenin has transcriptional activity (Figure 1(d)). While in healthy tissues, TL1A immunoreactivity was weak, while the majority of UC and CD patients displayed increased expression of TL1A in their colon tissues (Figure 1(d)). The mean optical density values of TL1A in the UC and CD groups were nearly 2-fold higher than that in the control group (Figure 1(d)). Next, the expression of epithelial cell marker E-cadherin, and myofibroblast markers FPS1 and *α*-SMA, was also assessed by immunohistochemical staining. E-cadherin expression stained more strongly in normal intestinal tissues than in UC and CD tissues (Figure 1(d)). In contrast, positive immunostaining for *α*-SMA and FPS1 exhibited increased intensity in UC and CD intestinal tissues. Altogether, these results confirm the appearance of EMT in IBD colon tissues (Figure 1(d)). Furthermore, the Pearson correlation and linear regression analyses were applied to explore the relationship between TL1A and EMT-related markers, which demonstrated that TL1A expression is negatively associated with E-cadherin expression (Figure 1(e)), while it is positively associated with nuclear localization of *β*-catenin, *α*-SMA, and FSP1 expression levels and fibrosis area (Figure 1(e)). Therefore, TL1A is likely to be involved in the EMT process in intestinal fibrosis.

### 3.3. Mice with High Expression of TL1A after DSS Induction Had More Severe Intestinal Inflammation

**Figure 2:**
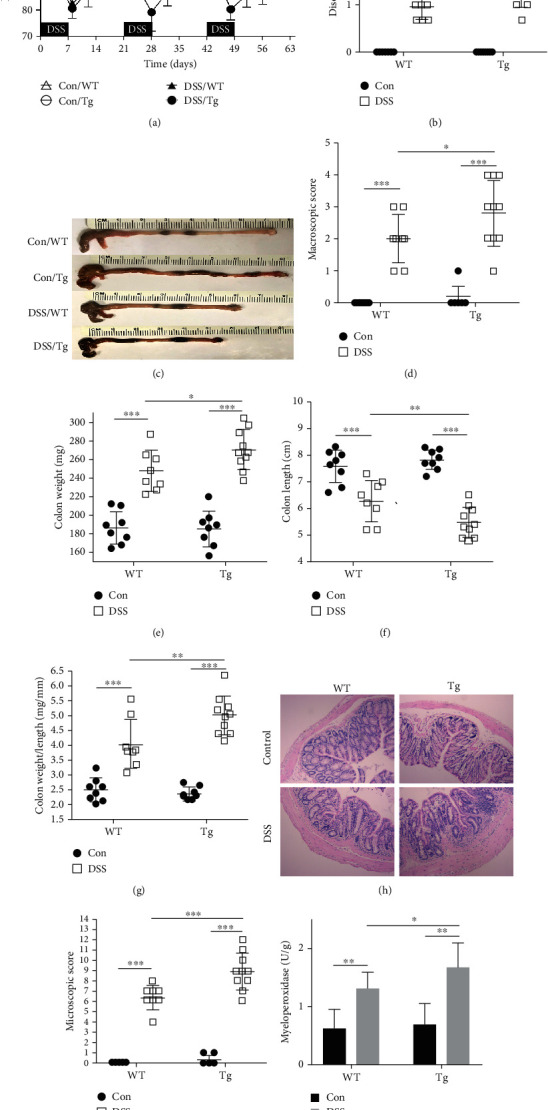
TL1A promotes colon inflammation in the DSS-induced intestinal fibrosis model. Transgenic (Tg) and wildtype (WT) mice were subjected to 2.0% dextran sulfate sodium (DSS) to generate an intestinal fibrosis model. There were four study groups: control/WT group (*n* = 8), control/Tg group (*n* = 8), DSS/WT group (*n* = 8), and DSS/Tg group (*n* = 10). (a) The percent change in body weight of mice during modeling. (b) Postmodeling disease activity index (DAI) based on body weight, stool characteristics, and fecal occult blood of mice. (c, d) Macroscopic damage and score. (e) Weight of colon. (f) Length of colon. (g) Colon weight/length ratio. (h) Representative images of H&E-stained colonic tissue sections of WT and Tg mice (100x). (i) Microscopic damage score. (j) Myeloperoxidase (MPO) activity. Data were given as mean ± standard deviation (SD). As compared to the control group: ^∗^*P* < 0.05, ^∗∗^*P* ≤ 0.01, and ^∗∗∗^*P* ≤ 0.001.

To further determine the contribution of TL1A in colonic inflammation, we divided WT mice and Tg mice each into two groups to administrate normal drinking water or 2% DSS as described above. After nine weeks, DAI scores and histopathological indicators were evaluated. During modeling, after drinking 2.0% DSS, the mice showed soft or loose, and visibly bloody stools, and a fluctuating decline in body weight, especially in the DSS/Tg group (Figure 2(a)). Meanwhile, the DAI score, which is performed based on mouse body weight, stool characteristics, and fecal occult blood increased more in the DSS/Tg group than in the DSS/WT group (Figure 2(b)). After the mice were euthanized, the colon was removed for macroscopic damage scoring. The control group (fed water without DSS) had no edema in the colon, while the intestinal mucosa of the DSS group showed hyperemic and edematous mucosa with thickening of the intestinal wall (Figure 2(c)). The macroscopic damage score in the DSS/Tg group was significantly higher than that in the DSS/WT group (Figure 2(d)). The DSS/Tg group had shorter colon length (Figure 2(f)), heavier colon weight (Figure 2(e)), and therefore, higher colon weight-to-length ratio (Figure 2(g)) than the DSS/WT group. These differences were statistically significant. H&E staining displayed that the colonic mucosal epithelium of the control group was intact and the glands were neatly arranged. However, in the DSS group, the colonic mucosal epithelium was destroyed, goblet cells were reduced or even missing, and neutrophils and lymphocytes had infiltrated the tissue (Figure 2(h)). The colonic microscopic damage score was significantly higher in the DSS/Tg group than that in the DSS/WT group (Figure 2(i)). MPO activity in the colon tissue reflects infiltration of inflammatory cells into the intestinal tissue. Compared with the DSS/WT group, MPO content was significantly increased in the DSS/Tg group (Figure 2(j)). The above results show that Tg mice with a high expression of TL1A are susceptible to inflammation in the colon.

### 3.4. Mice with High Expression of TL1A Had More Severe Intestinal Fibrosis

**Figure 3:**
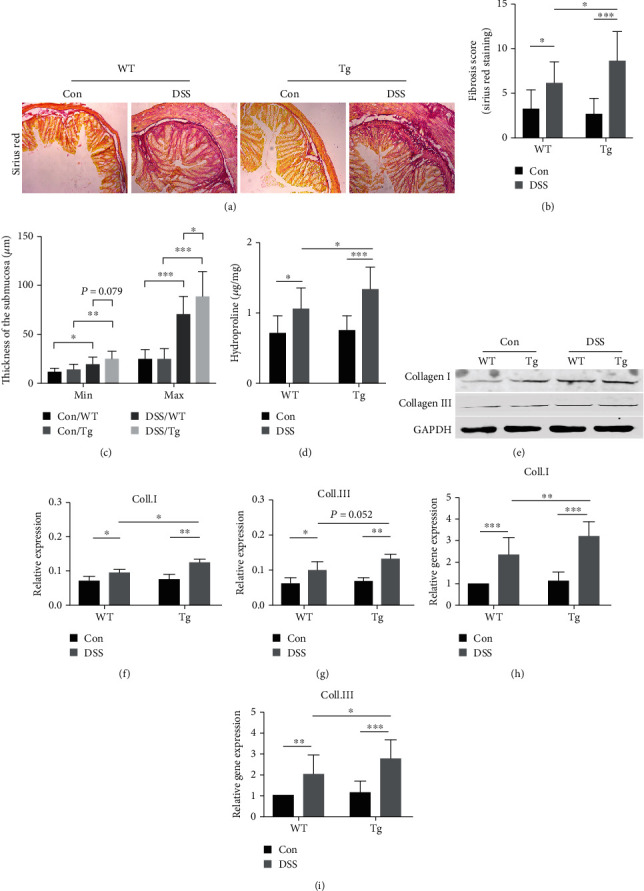
TL1A promotes colon fibrosis in the DSS-induced intestinal fibrosis model. (a) Sirius red staining of collagen deposition in colon from wild type and transgenic (Tg) mice overexpressing TL1A (100x). (b) Fibrosis scores reflect fibrotic alterations of colonic sections. (c) The relative minimal (min) and maximal (max) width of submucosa in mice. (d) Hydroxyproline content. (e–g) Western blot analysis of collagen I and collagen III in colonic tissues normalized with GAPDH. (h, i) RT-PCR detection of collagen I and collagen III mRNA. Data were given as mean ± standard deviation (SD). As compared to the control group: ^∗^*P* < 0.05, ^∗∗^*P* ≤ 0.01, and ^∗∗∗^*P* ≤ 0.001.

As previously reported, TL1A promoted intestinal fibrosis [22]. Sirius red staining showed that mice in the DSS group had a substantial amount of collagen deposition in colon tissues, especially in the DSS/Tg group (Figure 3(a)). Fibrotic alterations were quantified based on the fibrosis score according to the Supplementary Table 1. The DSS group showed a higher fibrosis score than the control group, and the DSS/Tg group had the highest score (Figure 3(b)). Compared with control group, the DSS group exhibited a significant increase in the minimum and maximum thickness of the submucosa (Figure 3(c)). The amount of hydroxyproline, one of the main components of collagen tissue, was significantly higher in DSS/Tg mice than in DSS/WT mice (Figure 3(d)). Collagen I and collagen III, components of ECM, are the main collagen fibers deposited when intestinal fibrosis occurs. Protein and mRNA levels of collagen I were greater in DSS/Tg mice than in DSS/WT mice (Figures 3(e), 3(f), and 3(h)) as were the mRNA levels of collagen III (Figure 3(i)). The protein levels of collagen III trended higher in the DSS/Tg group than in the DSS/WT group; however, this difference was not statistically significant (Figures 3(e) and 3(g)).

### 3.5. TL1A Promotes EMT *In Vivo*

**Figure 4:**
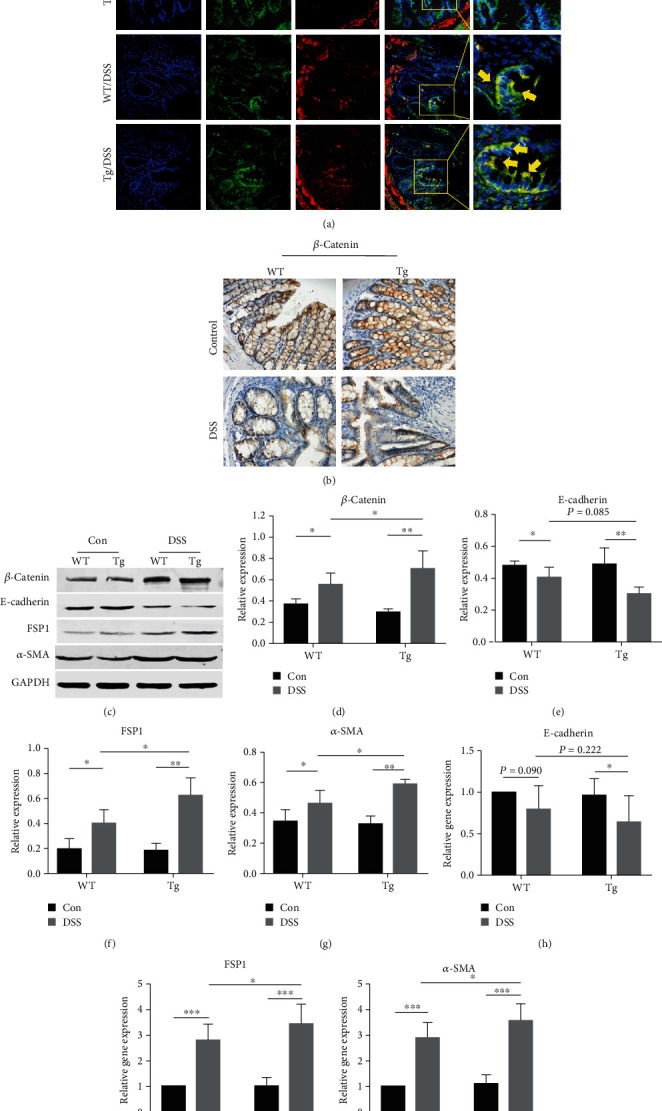
TL1A promotes EMT in the DSS-induced intestinal fibrosis model. (a) Immunofluorescence staining of colon tissue sections from wild type (WT) and transgenic (Tg) mice probed with antibodies against E-cadherin (green) and FSP1 (red) (400x). E-cadherin^+^FSP1^+^ cells (yellow; reflects EMT). Nuclei are stained with DAPI (blue). (b) Immunohistochemical staining of *β*-catenin in colon tissues of mice groups (400x). (c–g) Western blot analysis of total protein of *β*-catenin, E-cadherin, *α*-SMA, and FSP1 in intestinal tissue, normalized with GAPDH. (h–j) RT-PCR analysis of E-cadherin, *α*-SMA, and FSP1 mRNA levels in the indicated groups. Data were given as mean ± standard deviation (SD). As compared to the control group: ^∗^*P* < 0.05, ^∗∗^*P* ≤ 0.01, and ^∗∗∗^*P* ≤ 0.001.

EMT is a dynamic process during which cells can simultaneously express epithelial and mesenchymal markers, e.g., E-cadherin and FSP1, respectively. Therefore, the colocalization of these proteins was assessed. Immunofluorescence costaining showed that green fluorescent E-cadherin was apparently decreased in DSS mice, while red fluorescent FSP1 was apparently increased in DSS mice. Moreover, yellow fluorescence, representing E-cadherin and FSP1 colocalized expression, was stronger in DSS/Tg mice than in DSS/WT mice, indicating that TL1A participates in the EMT process (Figure 4(a)). Furthermore, immunohistochemical staining showed that *β*-catenin, which is a well-established marker for the onset of EMT, is no longer a membrane associated in the DSS group but localized in the cytoplasm or even in the nucleus (Figure 4(b)). Next, the levels of protein and mRNA expression of epithelial marker E-cadherin and interstitial markers FSP1 and *α*-SMA were determined by Western blot analysis and RT-PCR. There was no statistically significant difference in the protein and mRNA expression levels of E-cadherin between the DSS/WT group and the DSS/Tg group (Figures 4(c), 4(e), and 4(h)), while the total protein level of *β*-catenin, FSP1, and *α*-SMA expression levels were statistically increased in the DSS/WT group, especially in the DSS/Tg group (Figures 4(c), 4(d), 4(f), 4(g), 4(i), and 4(j)).

### 3.6. TL1A May Affect EMT through TGF-*β*/Smad3 Pathways in Patients with IBD

**Figure 5:**
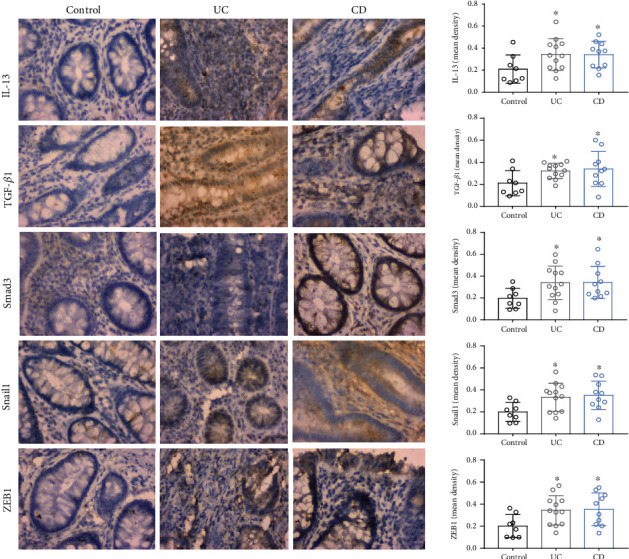
IL-13, TGF-*β*1, Smad3, and EMT-related transcriptional factor expression in IBD colon tissues. Immunohistochemical staining of IL-13, TGF-*β*1, Smad3, ZEB1, and Snail1 in intestinal tissues of controls, ulcerative colitis (UC), and Crohn's disease (CD) patients (400x). Data were given as mean ± SD. As compared to the control group: ^∗^*P* < 0.05, ^∗∗^*P* ≤ 0.01, and ^∗∗∗^*P* ≤ 0.001.

To explore the possible mechanism of TL1A regulation of EMT, colonic sections from control patients and patients with IBD were assessed for proteins of the TGF-*β*1/Smad3 pathway and EMT-related transcripts by immunohistochemical staining. Fibrogenic factor IL-13, TGF-*β*1, and Smad3 were found to be increased in UC and CD groups compared with the control group. The TGF-*β1*/Smad3 pathway is a classical way involved in EMT. The SMAD complex enters the nucleus and inhibits or activates target genes, such as Snail, ZEB, and basic helix-loop-helix (bHLH). Therefore, we tested the expression of ZEB1 and Snail1 and found that compared with control group, these two proteins were expressed higher in the UC and CD groups (Figure 5).

### 3.7. TL1A Induces EMT through TGF-*β*/Smad3 Pathways in the DSS-Induced Intestinal Fibrosis Model

**Figure 6:**
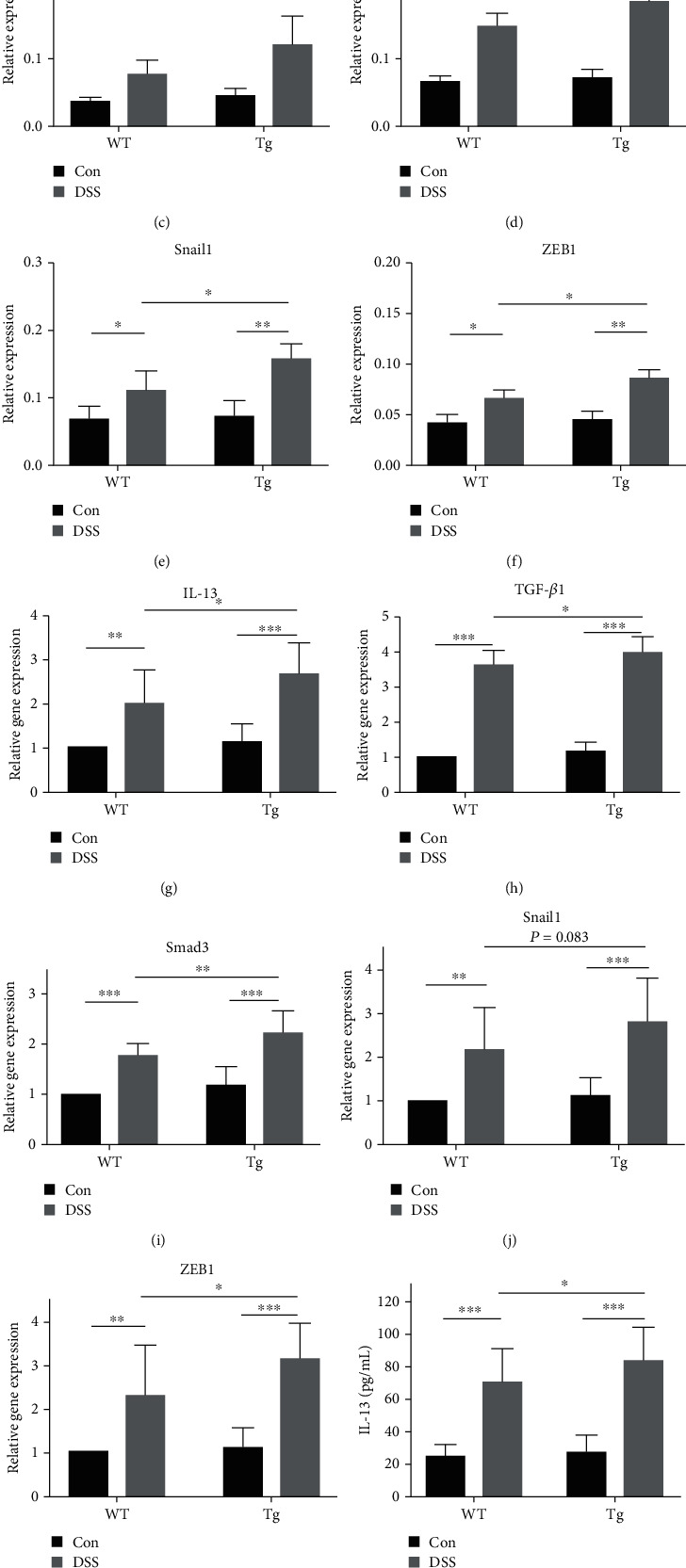
TL1A promotes epithelial to mesenchymal transition (EMT) via IL-13 and the TGF-*β*1/Smad3 pathway in DSS-induced colitis-related intestinal fibrosis mouse model. (a–f) Western blot analysis of IL-13, TGF-*β*1, Smad3, ZEB1, and Snail1 in intestinal tissues from previously described groups. (g–k) RT-PCR analysis of IL-13, TGF-*β*1, Smad3, ZEB1, and Snail1 mRNA levels in mice treated as described above. (l, m) ELISA showed expressions of IL-13 and TGF-*β*1. (n) Serum TGF-*β*1 was associated with IL-13 expression. As compared to the control group: ^∗^*P* < 0.05, ^∗∗^*P* ≤ 0.01, and ^∗∗∗^*P* ≤ 0.001.

Western blot analysis and RT-PCR were performed to explore the effects of TL1A in the DSS-induced intestinal fibrosis model. The TGF-*β*1 protein levels were greater in DSS/Tg mice than in DSS/WT mice; however, the difference was not statistically significant (Figures 6(a) and 6(c)), while IL-13, Smad3, Snail1, and ZEB1 protein expression was significantly higher in DSS/Tg mice than in DSS/WT mice (Figures 6(a), 6(b), 6(d), 6(e), and 6(f)). The mRNA levels of IL-13, Smad3, TGF-*β*1, and ZEB1 were significantly greater in DSS/Tg mice than that in DSS/WT mice (Figures 6(g), 6(h), 6(i), and 6(k)), while there was no statistically significant difference in mRNA levels of Snail1 between DSS/Tg mice and DSS/WT mice (Figure 6(j)). The presence of IL-13 and TGF-*β*1 in serum from the DSS-induced intestinal fibrosis mouse model was detected by ELISA. The levels of IL-13 and TGF-*β*1 in circulating serum of DSS/Tg mice were increased compared with that of DSS/WT mice (Figures 6(l) and 6(m)). Furthermore, a positive correlation between IL-13 and TGF-*β*1 in the serum was observed (Figure 6(n)).

### 3.8. Inhibition of Intestinal Fibrosis by BMP-7 or Anti-TL1A Antibody *In Vitro*

**Figure 7:**
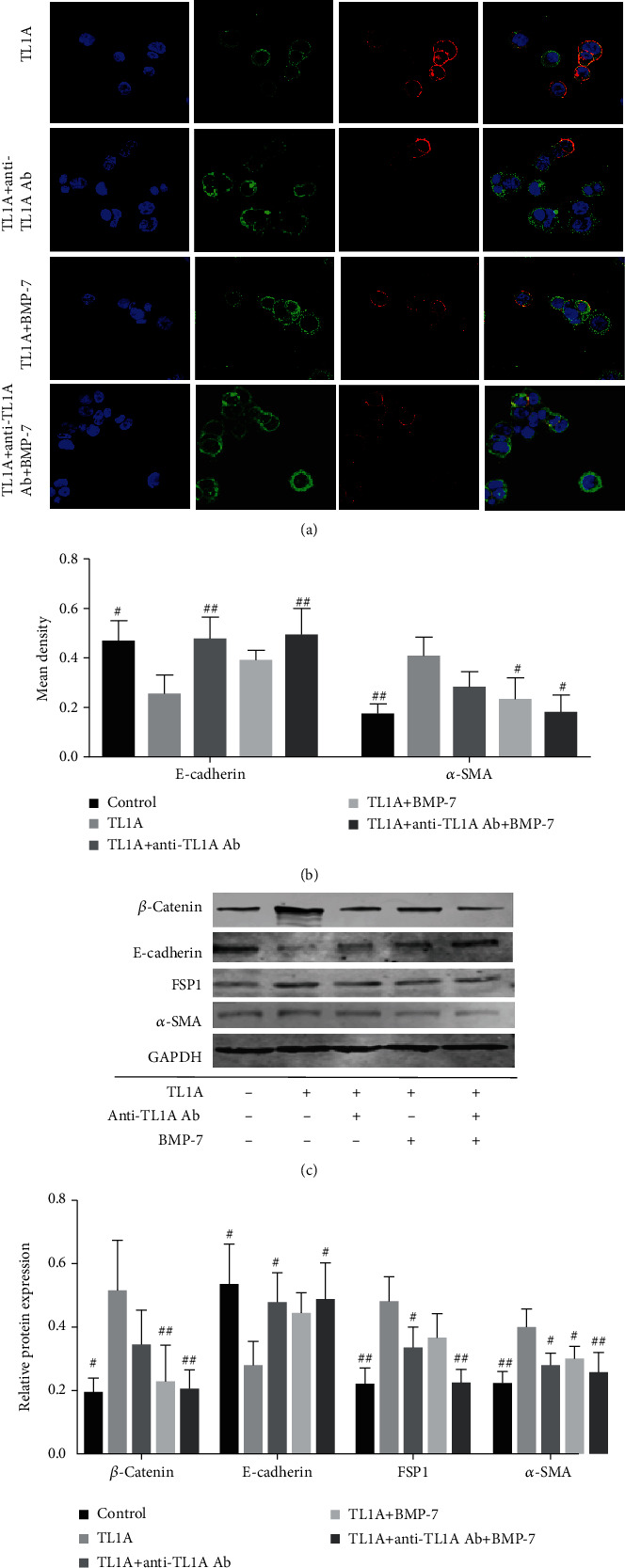
Inhibition of TL1A in intestinal epithelial cells *in vitro* using anti-TL1A antibodies and BMP-7. HT-29 cells were exposed to TL1A, anti-TL1A antibodies, and/or BMP-7, respectively. (a, b) The cells were probed with fluorescently labeled antibodies against E-cadherin (red) and FSP1 (green); E-cadherin and FSP1 colocalization (yellow) (1000x) and the mean density of E-cadherin and FSP1 were detected by IPP software. (c, d) Western blot analysis of *β*-catenin, E-cadherin, *α*-SMA, and FSP1 in different groups of HT-29 cells. ^∗^*P* < 0.05, ^∗∗^*P* < 0.01, and ^∗∗∗^*P* < 0.001 versus TL1A-treated cells. Data were given as mean ± standard deviation (SD). ^#^*P* < 0.05, ^##^*P* < 0.01, and ^###^*P* < 0.001 versus TL1A-treated cells.

EMT was induced in human HT-29 cells under the stimulation of TL1A. HT-29 cells in control medium without TL1A had intense E-cadherin expression and FSP1 labeling (Figures 7(a) and 7(b)). In contrast, upon incubation with TL1A, HT-29 cells lost E-cadherin expression, and FSP1 expression increased. The addition of BMP-7 or anti-TL1A antibody to the medium prevented EMT induced by TL1A. Additionally, the inhibitory effects of BMP-7 and anti-TL1A antibody *in vitro* were highly similar. The expression levels of *β*-catenin, E-cadherin, FSP1, and *α*-SMA were compared using Western blot analysis (Figures 7(c) and 7(d)). Compared with the control group, the expression of E-cadherin was decreased in TL1A group but was upregulated when BMP-7 or anti-TL1A antibodies were added. In contrast, the expression of *β*-catenin, FSP1, and *α*-SMA was decreased after the addition of BMP-7 or anti-TL1A antibodies.

## 4. Discussion

Intestinal stenosis and obstruction caused by intestinal fibrosis are still intractable problems in the treatment of IBD. Myofibroblasts are key effector cells in the formation of intestinal fibrosis [27]. Multiple studies have stressed that EMT is one of the important sources of intestinal myofibroblasts [12, 28, 29]. However, there are still many unknowns that need to be investigated. This study provides strong evidence for the involvement of TL1A in intestinal fibrosis both *in vitro* and *in vivo*. The abnormal accumulation of TL1A in intestinal mucosa of patients with IBD is related to the degree of fibrosis and EMT-related markers. In addition, TL1A participates in intestinal fibrosis by promoting the secretion of inflammatory factors such as IL-13 and TGF-*β*1, and by activating the expression of EMT-related transcription factors via the TGF-*β*1/Smad3 pathway. TL1A can also directly induce EMT *in vitro*, and the process can be effectively inhibited by anti-TL1A antibodies or BMP-7. These findings underline the potential role of TL1A in EMT and provide evidence for antifibrosis treatment in the future.

A growing body of evidence suggests that TL1A is closely linked to the process of inflammation and fibrosis diseases [30–32]. TL1A is a member of the TNF family, which can bind to death receptor 3 (DR3) [33], and can enhance T cell proliferation and cytokine production [34]. A previous study demonstrated that overexpression of TL1A in myeloid cells can aggravate liver fibrosis by macrophage recruitment and cytokine secretion [35]. Moreover, TL1A can promote intestinal fibrosis by disrupting immune responses [22] or intestinal flora [18]. It has been reported that transgenic mice with TL1A overexpression develop spontaneous ileitis and inflammation and fibrosis of proximal colitis [19]. GWAS support a role for TL1A in the process of IBD. In this study, serum inflammation indicators and intestinal mucosal inflammation were more severe in patients with IBD, and large amounts of collagen fibers were deposited in the intestinal mucosa of these patients. Immunohistochemical staining showed that TL1A expression in the UC and CD groups was significantly increased compared with the control group. The expression of TL1A was positively associated with fibrosis area and with the expression of *β*-catenin, FSP1, and *α*-SMA, but was negatively associated with expression of E-cadherin. These results confirm that TL1A may participate in the process of intestinal fibrosis through EMT. Furthermore, similar results were obtained in animal experiments. Wild-type mice and transgenic mice with high TL1A expression were given DSS to construct a chronic colitis-related fibrosis model. The DSS groups exhibited more severe intestinal inflammation and collagen deposition, and the protein and mRNA expressions of collagen I and collagen III were increased. These findings were more pronounced in the DSS/Tg group, indicating that the high expression of TL1A increased susceptibility to intestinal inflammation and fibrosis.

EMT is the process of tissue repair and healing, in which epithelial cells lose polarity and intercellular adhesions to obtain interstitial markers and transform into mesenchymal stem cells [36]. Flier et al. [11] used immunofluorescence staining to evaluate the colocalization of FSP1 and E-cadherin, and found that E-cadherin^+^FSP1^+^ cells were detected in mice treated with 2,4,6-trinitrobenzene sulfonic acid (TNBS). The same conclusion can be obtained in our research. The epithelial marker E-cadherin was significantly decreased in DSS/Tg mice compared with DSS/WT mice, and the interstitial marker FSP1 was significantly increased in DSS/Tg mice compared with DSS/WT mice, confirming that EMT is involved in DSS-induced intestinal fibrosis.

Many studies suggest that TL1A promotes the development of inflammation by inducing Th2/IL-13 mucosal responses, which are currently considered a main cause of colitis. Mice with constitutive expression of TL1A in lymphoid or myeloid cells exhibit spontaneous ileitis [19, 32, 37]. The upregulation of activated T cells and regulatory Foxp3^+^CD4^+^ T cells exacerbates intestinal inflammation related to the mucosal response of Th2 cells [38]. Expression of IL-13 was increased in TL1A transgenic mice, and blocking IL-13 release alleviates the severity of ileitis [19, 32, 37]. In our study, the protein and mRNA expression levels of IL-13 were significantly higher in DSS/Tg mice than in DSS/WT mice. Therefore, we concluded that TL1A affects the intestinal fibrosis process by promoting the secretion of the fibrotic factor IL-13.

The fourth ECCO guidelines state that IL-13 activates the TGF-*β*1/Smad3 pathway as the central process in intestinal fibrosis formation [21]. It is reported that IL-4 and IL-13 can synergize with high glucose to promote the expression of TGF-*β*1, FN, and collagen I in cultured HK-2 cells, indicating that the secretion of Th2 cytokines (IL-4 and IL-13) and the upregulated expression of TGF-*β*1 are key factors in the development of renal fibrosis [39]. Another study showed that IL-13 induces fibrosis through the activation of TGF-*β*1, and the synergistic effect of IL-13 and TGF-*β*1 may increase the expression of eotaxin in human fibroblast [40]. It has also been reported that IL-13 expression could lead to the proliferation of bronchial epithelial cells via the production of TGF-*α* and activation of the epidermal growth factor receptor (EGFR) [41]. In the current study, IL-13 and TGF-*β*1 levels were significantly upregulated in the serum of DSS mice (most dramatically in DSS/Tg mice) compared with those of control mice. This positive correlation indicates that IL-13 and TGF-*β*1 may be involved in EMT of intestinal fibrosis.

EMT involves a large number of cell signaling pathways and complex gene regulation processes, including classical TGF-*β*-dependent and TGF-*β*-independent pathways. TGF-*β* signals by binding to its receptor to activate the Smad complex, which can translocate to the nucleus and regulate the expression of target genes, such as Snail, ZEB, and bHLH. In clinical studies, the expression of Slug in inflammatory mucosa and fibrotic tissue of UC and CD patients was shown to be increased [42], and the expression of Snail was also increased in relation to fistula formation of IBD [43]. In the current study, the protein and mRNA levels of EMT-related transcription factors Snail1 and ZEB1 were detected by immunohistochemical staining, Western blot analysis, and RT-PCR, respectively. It was found that Snail1 and ZEB1 expression was higher in DSS mice, especially in DSS/Tg mice, than in controls, indicating that EMT is involved in intestinal fibrosis.

As previously reported, the application of anti-TL1A antibodies reduce the expression of *α*-SMA and vimentin, and are even capable of reversing intestinal fibrosis by suppressing the TGF-*β1*/Smad3 pathway in adoptively transferred T-cell colitic mice [22]. BMP-7 is a member of the transforming growth factor *β* (TGF-*β*) superfamily and has antifibrotic effects in the liver through inhibition of the TGF-*β1*/Smad pathway [44]. In mice treated with TNBS, colocalization of E-cadherin and FSP1 was significantly decreased when mice received concomitant BMP-7 treatment [11]. In our study, immunofluorescence staining of HT-29 cells stimulated with TL1A showed that the expression of E-cadherin decreased, while the expression of FSP1 increased. This process was blocked by the application of either anti-TL1A antibody or BMP-7, and the inhibitory effect was maximized by using both *in vitro*. This could offer as a novel strategy for the cure or reversal of intestinal fibrosis.

In summary, we conclude that increased TL1A levels correlate with IBD-associated intestinal fibrosis. Accumulation of TL1A induces EMT and collagen fiber production in intestinal epithelial cells through an IL-13- and TGF-*β*1/Smad3 signaling pathway-mediated process. Inhibition of TL1A expression, therefore, may provide an effective strategy for relieving intestinal fibrosis.

## Figures and Tables

**Table 1:** Patient characteristics of human samples.

	Controls (*n* = 8)	UC (*n* = 12)	CD (*n* = 10)
Mean age (year, range)	45.5 ± 9.68	40.25 ± 13.05	37.40 ± 12.55
Females	4 (50%)	6 (50%)	6 (60%)
Males	4 (50%)	6 (50%)	4 (40%)
Mean disease duration (year, range)	0	6.25 ± 2.14	6.80 ± 2.25
Disease activity			
No	8 (100%)	0 (0%)	0 (0%)
Low	0	3 (25%)	3 (30%)
Moderate	0	5 (41.67%)	5 (50%)
Strong	0	4 (33.33%)	2 (20%)

UC: ulcerative colitis; CD: Crohn's disease. Disease activity based on modified Mayo score. Data are expressed as mean ± SD.

**Table 2:** Inflammation markers in IBD patients and controls.

	Control (*n* = 8)	UC (*n* = 12)	CD (*n* = 10)
WBC (/mm^3^ × 10^3^)	5.11 ± 0.85	7.16 ± 1.88^∗^	6.95 ± 1.82^∗^
hsCRP (mg/L)	1.35 ± 1.44	28.43 ± 34.23^∗^	24.15 ± 22.61^∗^
ESR (mm/h)	4.50 ± 2.14	23.41 ± 14.03^∗∗^	20.90 ± 12.66^∗∗^
HGB (g/L)	134.50 ± 13.44	119.42 ± 19.51	128.10 ± 19.30
NE (/mm^3^ × 10^3^)	3.01 ± 0.60	4.45 ± 1.58^∗^	3.54 ± 1.46
PLT	240.05 ± 61.72	270.61 ± 78.08^∗^	355.53 ± 120.73
ALB	44.8 ± 52.89	36.00 ± 6.95^∗∗^	37.53 ± 4.43^∗∗^

WBC: white blood cell; hsCRP: high-sensitivity C-reactive protein; ESR: erythrocyte sedimentation rate; HGB: hemoglobin; NE: neutrophil; PLT: blood platelet; ALB: albumin. Data are expressed as mean ± SD. As compared to the control group: ^∗^*P* < 0.05; ^∗∗^*P* ≤ 0.01; ^∗∗∗^*P* ≤ 0.001.

## Data Availability

The data used to support the findings of this study are available from the author upon request.

## Supplementary Materials

Supplementary Materials Supplementary Table 1 Criteria for Histologic Fibrosis Score of Intestine. Supplementary Table 2 Primers used for qRT-PCR analysis. Supplementary Figure 1 The identification of the LCK-CD2-TL1A-GFP transgenic mouse. Supplementary Figure 2 Effect of TL1A on HT-29 cell viability.

## References

[1] Pakshir P., Hinz B. (2018). The big five in fibrosis: macrophages, myofibroblasts, matrix, mechanics, and miscommunication. *Matrix Biology*.

[2] Lenti M. V., Di S. A. (2019). Intestinal fibrosis. *Molecular Aspects of Medicine*.

[3] Thia K. T., Sandborn W. J., Harmsen W. S., Zinsmeister A. R., Loftus E. V. (2010). Risk Factors Associated With Progression to Intestinal Complications of Crohn's Disease in a Population-Based Cohort. *Gastroenterology*.

[4] Rieder F., Zimmermann E. M., Remzi F. H., Sandborn W. J. (2013). Crohn’s disease complicated by strictures: a systematic review. *Gut*.

[5] Rieder F., Fiocchi C., Rogler G. (2017). Mechanisms, management, and treatment of fibrosis in patients with inflammatory bowel diseases. *Gastroenterology*.

[6] Gordon I. O., Agrawal N., Goldblum J. R., Fiocchi C., Rieder F. (2014). Fibrosis in ulcerative colitis: mechanisms, features, and consequences of a neglected problem. *Inflammatory Bowel Diseases*.

[7] Gordon I. O., Agrawal N., Willis E. (2018). Fibrosis in ulcerative colitis is directly linked to severity and chronicity of mucosal inflammation. *Alimentary Pharmacology & Therapeutics*.

[8] Wu F., Shao Q., Hu M. (2020). Wu-Mei-Wan ameliorates chronic colitis-associated intestinal fibrosis through inhibiting fibroblast activation. *Journal of Ethnopharmacology*.

[9] Ortiz-Masià D., Salvador P., Macias-Ceja D. C. (2020). WNT2b activates epithelial-mesenchymal transition through FZD4: relevance in penetrating Crohn’s disease. *Journal of Crohn's & Colitis*.

[10] Nieto M. A., Huang R. Y., Jackson R. A., Thiery J. P. (2016). EMT: 2016. *Cell*.

[11] Flier S. N., Tanjore H., Kokkotou E. G., Sugimoto H., Zeisberg M., Kalluri R. (2010). Identification of epithelial to mesenchymal transition as a novel source of fibroblasts in intestinal fibrosis. *The Journal of Biological Chemistry*.

[12] He S., Xue M., Liu C., Xie F., Bai L. (2018). Parathyroid hormone-like hormone induces epithelial-to-mesenchymal transition of intestinal epithelial cells by activating the runt-related transcription factor 2. *The American Journal of Pathology*.

[13] Scharl M., Huber N., Lang S., Fürst A., Jehle E., Rogler G. (2015). Hallmarks of epithelial to mesenchymal transition are detectable in Crohn’s disease associated intestinal fibrosis. *Clinical and Translational Medicine*.

[14] Yang S. K., Hong M., Zhao W. (2014). Genome-wide association study of Crohn’s disease in Koreans revealed three new susceptibility loci and common attributes of genetic susceptibility across ethnic populations. *Gut*.

[15] Arimura Y., Isshiki H., Onodera K. (2014). Characteristics of Japanese inflammatory bowel disease susceptibility loci. *Journal of Gastroenterology*.

[16] Hong S. N., Park C., Park S. J. (2016). Deep resequencing of 131 Crohn’s disease associated genes in pooled DNA confirmed three reported variants and identified eight novel variants. *Gut*.

[17] Yang M., Jia W., Wang D. (2019). Effects and mechanism of constitutive TL1A expression on intestinal mucosal barrier in DSS-induced colitis. *Digestive Diseases and Sciences*.

[18] Jacob N., Jacobs J. P., Kumagai K. (2018). Inflammation-independent TL1A-mediated intestinal fibrosis is dependent on the gut microbiome. *Mucosal Immunology*.

[19] Meylan F., Song Y. J., Fuss I. (2011). The TNF-family cytokine TL1A drives IL-13-dependent small intestinal inflammation. *Mucosal Immunology*.

[20] Giuffrida P., Caprioli F., Facciotti F., di Sabatino A. (2019). The role of interleukin-13 in chronic inflammatory intestinal disorders. *Autoimmunity Reviews*.

[21] Latella G., Rogler G., Bamias G. (2014). Results of the 4th scientific workshop of the ECCO (I): pathophysiology of intestinal fibrosis in IBD. *Journal of Crohn's & Colitis*.

[22] Li H., Song J., Niu G. (2018). TL1A blocking ameliorates intestinal fibrosis in the T cell transfer model of chronic colitis in mice. *Pathology, Research and Practice*.

[23] Barrett R., Zhang X., Koon H. W. (2012). Constitutive TL1A expression under colitogenic conditions modulates the severity and location of gut mucosal inflammation and induces fibrostenosis. *The American Journal of Pathology*.

[24] Aranda R., Sydora B. C., PL M. A. (1997). Analysis of intestinal lymphocytes in mouse colitis mediated by transfer of CD4+, CD45RBhigh T cells to SCID recipients. *Journal of Immunology*.

[25] Theiss A. L., Fuller C. R., Simmons J. G., Liu B., Sartor R. B., Lund P. K. (2005). Growth hormone reduces the severity of fibrosis associated with chronic intestinal inflammation. *Gastroenterology*.

[26] Scheibe K., Kersten C., Schmied A. (2019). Inhibiting interleukin 36 receptor signaling reduces fibrosis in mice with chronic intestinal inflammation. *Gastroenterology*.

[27] Li C., Kuemmerle J. F. (2020). The fate of myofibroblasts during the development of fibrosis in Crohn's disease. *Journal of Digestive Diseases*.

[28] Pierdomenico M., Palone F., Cesi V. (2018). Transcription factor ZNF281: a novel player in intestinal inflammation and fibrosis. *Frontiers in Immunology*.

[29] Xu X., Sun S., Xie F. (2017). Advanced oxidation protein products induce epithelial-mesenchymal transition of intestinal epithelial cells via a PKC *δ*-mediated, redox-dependent signaling pathway. *Antioxidants & Redox Signaling*.

[30] Tougaard P., Martinsen L. O., Zachariassen L. F. (2019). TL1A aggravates cytokine-induced acute gut inflammation and potentiates infiltration of intraepithelial natural killer cells in mice. *Inflammatory Bowel Diseases*.

[31] Yuan Z. C., Wang J. M., Su L. C., Xu W. D., Huang A. F. (2019). Gene polymorphisms and serum levels of TL1A in patients with rheumatoid arthritis. *Journal of Cellular Physiology*.

[32] Shih D. Q., Barrett R., Zhang X. (2011). Constitutive TL1A (TNFSF15) expression on lymphoid or myeloid cells leads to mild intestinal inflammation and fibrosis. *PLoS One*.

[33] Migone T. S., Zhang J., Luo X. (2002). TL1A is a TNF-like ligand for DR3 and TR6/DcR3 and functions as a T cell costimulator. *Immunity*.

[34] Meylan F., Richard A. C., Siegel R. M. (2011). TL1A and DR3, a TNF family ligand-receptor pair that promotes lymphocyte costimulation, mucosal hyperplasia, and autoimmune inflammation. *Immunological Reviews*.

[35] Guo J., Luo Y., Yin F. (2019). Overexpression of Tumor Necrosis Factor-Like Ligand 1 A in Myeloid Cells Aggravates Liver Fibrosis in Mice. *Journal of Immunology Research*.

[36] Yang Z., Xie Q., Chen Z. (2019). Resveratrol suppresses the invasion and migration of human gastric cancer cells via inhibition of MALAT1-mediated epithelial-to-mesenchymal transition. *Experimental and Therapeutic Medicine*.

[37] Taraban V. Y., Slebioda T. J., Willoughby J. E. (2011). Sustained TL1A expression modulates effector and regulatory T-cell responses and drives intestinal goblet cell hyperplasia. *Mucosal Immunology*.

[38] Valatas V., Kolios G., Bamias G. (2019). TL1A (TNFSF15) and DR3 (TNFRSF25): a co-stimulatory system of cytokines with diverse functions in gut mucosal immunity. *Frontiers in Immunology*.

[39] Liu C., Qin L., Ding J. (2019). Group 2 innate lymphoid cells participate in renal fibrosis in diabetic kidney disease partly via TGF-*β*1 signal pathway. *Journal Diabetes Research*.

[40] Koga H., Miyahara N., Fuchimoto Y. (2013). Inhibition of neutrophil elastase attenuates airway hyperresponsiveness and inflammation in a mouse model of secondary allergen challenge: neutrophil elastase inhibition attenuates allergic airway responses. *Respiratory Research*.

[41] Allahverdian S., Harada N., Singhera G. K., Knight D. A., Dorscheid D. R. (2008). Secretion of IL-13 by airway epithelial cells enhances epithelial repair via HB-EGF. *American Journal of Respiratory Cell and Molecular Biology*.

[42] Zidar N., Boštjančič E., Jerala M. (2016). Down-regulation of microRNAs of the miR-200 family and up-regulation of Snail and Slug in inflammatory bowel diseases—hallmark of epithelial-mesenchymal transition. *Journal of Cellular and Molecular Medicine*.

[43] Scharl M., Weber A., Fürst A. (2011). Potential role for SNAIL family transcription factors in the etiology of Crohn’s disease-associated fistulae. *Inflammatory Bowel Diseases*.

[44] Zou G. L., Zuo S., Lu S. (2019). Bone morphogenetic protein-7 represses hepatic stellate cell activation and liver fibrosis via regulation of TGF-*β*/Smad signaling pathway. *World Journal of Gastroenterology*.

