# Supplementary material for: Effect and Mechanism of TL1A Expression on Epithelial-Mesenchymal Transition during Chronic Colitis-Related Intestinal Fibrosis
Source: Mediators Inflamm. 2021 Jun 25;2021:5927064. doi: 10.1155/2021/5927064 (PMC8253633; doi:10.1155/2021/5927064)
Supplement: Supplementary Materials — Supplementary Table 1 Criteria for Histologic Fibrosis Score of Intestine. Supplementary Table 2 Primers used for qRT-PCR analysis. Supplementary Figure 1 The identification of the LCK-CD2-TL1A-GFP transgenic mouse. Supplementary Figure 2 Effect of TL1A on HT-29 cell viability. [file 5927064.f1.doc]

**Supplementary materials**

**Supplementary Table 1 Criteria for Histologic Fibrosis Score of Intestine**

|  | Score | Description |
| --- | --- | --- |
| Fibrosis | 0 | No increased collagen deposition |
|  | 1 | Increased collagen deposition in submucosa |
|  | 2 | Increased collagen deposition in submucosa and mucosa |
|  | 3 | Increased collagen deposition in muscularis mucosa, submucosa, and mucosa; thickening, disorganization of the muscularis mucosa |
|  | 4 | Increased collagen deposition in muscularis propria, muscularis mucosa, submucosa, and mucosa |
|  | 5 | Increased collagen deposition throughout all layers including serosa |
| Percent involvement | 1 | 0–25% of section |
|  | 2 | 25–50% of section |
|  | 3 | 50–75% of section |
|  | 4 | 75–100% of section |

Supplementary Table 2 Primers used for qRT-PCR analysis

| Gene | Amplification primers | Amplifcation fragment (bp) |
| --- | --- | --- |
| GAPDH | Sense:5’ TCGTCCCGTAGACAAAATGG 3’  Antisense:5’ TTGAGGTCAATGAAGGGGTC3 3’ | 132 bp |
| α-SMA | Sense: 5'TGCTGTCCCTCTATGCCTCT 3'  Antisense: 5'GAAGGAATAGCCACGTCAG3' | 122 bp |
| Collagen I | Sense: 5' GCTGGAAAGGAAGGGATT 3'  Antisense: 5'GGGAGCACCAAGAAGACC 3' | 174 bp |
| Collagen III | Sense: 5' CCCACAGCCTTCTACACCT 3'  Antisense: 5' CCAGGGTCACCATTTCTC 3' | 108 bp |
| FSP1 | Sense: 5' CAGAAGGTGATGAGCAACT 3'  Antisense: 5' CGAAGAAGCCAGAGTAAGG 3' | 201 bp |
| E-cadherin | Sense: 5' GACTTAGAGATTGGCGAATAC 3'  Antisense: 5' GAGGATGGCAGGAACTTG 3' | 371 bp |
| TL1A | Sense:5’ CGGGGAGACGACCAAACAAG 3’  Antisense:5’ AAGGAGAACGTGGCCCCA 3’ | 160 bp |
| IL-13 | Sense:5’CAG TCC TGG CTC TTG CTT G 3’  Antisense:5’ CCA GGT CCA CAC TCC ATA CC 3’ | 157 bp |
| TGF-β1 | Sense:5’ AACTAAGGCTCGCCAGTCC 3’  Antisense:5’ GCGGTCCACCATTAGCAC 3’ | 182 bp |
| Smad3 | Sense:5’ CAGCCTGTTTCTGAGACCAC 3’  Antisense:5’ GCGATACACCACCTGTTAGTTC 3’ | 132 bp |
| Snail1 | Sense:5’ TGTAACAAGGAGTACCTCAG 3’  Antisense:5’ GCACTGGTATCTCTTCACA 3’ | 144 bp |
| ZEB1 | Sense:5’ TCGGAAGACAGAGAATGGA 3  Antisense:5’ AGTAGGAGTAGCGGTGATT 3’ | 132 bp |

**supplementary figure 1**

**
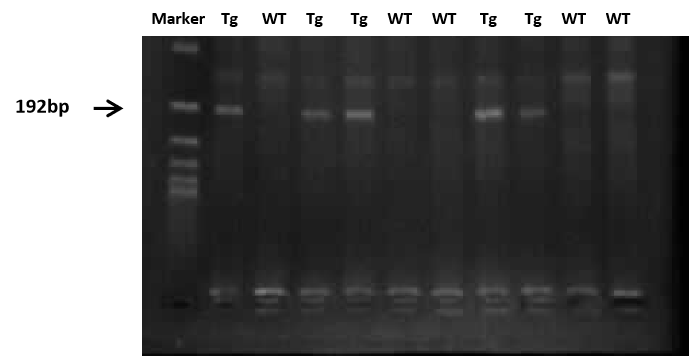
**

**Supplementary Figure 1** The identification of the LCK-CD2-TL1A-GFP transgenic mouse.

The PCR amplification of a 192 bp DNA fragment specific to *TL1A* located in Tg mice but not expressed in WT mice using the primers: 5’-GACTAACAAAGATGCCTGCCTGTGG-3’ and 5’-GCCATCCTTCTG CTGTCTTGGAGA-3’.

**Supplementary figure 2**


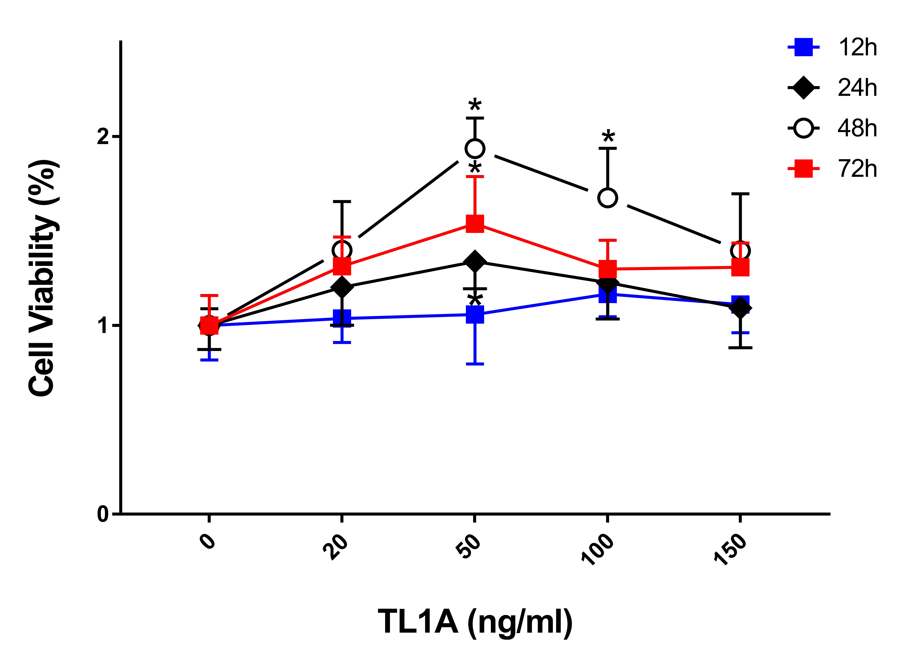


**Supplementary Figure 2** Effect of TL1A on HT-29 cell viability.

The viability of HT-29 cells after treatment with different concentrations of TL1A at 12, 24, 48 and 72 hours as tested by cell counting kit-8 (CCK-8). The data were expressed as mean ± SD. **P*<0.05 compared with that of non-TL1A-treated cells.
